# FT-IR Spectroscopy to Discriminate Old from New *Pseudomonas aeruginosa* Infections in People with Cystic Fibrosis

**DOI:** 10.3390/ijms27146452

**Published:** 2026-07-20

**Authors:** Martina Rossitto, Serena Raimondi, Valeria Fox, Vanessa Tuccio Guarna Assanti, Nour Essa, Maria Stefania Lepanto, Marco Cristiano, Venere Cortazzo, Marilena Agosta, Fabio Majo, Renato Cutrera, Carlo Federico Perno, Paola Bernaschi, Gianluca Vrenna

**Affiliations:** 1 Multimodal Laboratory Medicine, Bambino Gesù Children’s Hospital, IRCCS, 00165 Rome, Italy; martina.rossitto@opbg.net; 2 Microbiology and Diagnostic Immunology Unit, Bambino Gesù Children’s Hospital, IRCCS, 00165 Rome, Italy; serena.raimondi@opbg.net (S.R.); gianluca.vrenna@opbg.net (G.V.); 3 Pneumology and Cystic Fibrosis Unit, Bambino Gesù Children’s Hospital, IRCCS, 00165 Rome, Italy

**Keywords:** cystic fibrosis, Fourier-transform infrared spectroscopy, *Pseudomonas aeruginosa*, whole genome sequencing, modulator, eradication

## Abstract

Chronic *Pseudomonas aeruginosa* colonisation leads to lung deterioration and poor prognosis in people with cystic fibrosis (pwCF). Early and aggressive therapies can achieve *P. aeruginosa* eradication, which may recur later. Therefore, determining whether it has resisted therapy or has been newly acquired may guide subsequent treatment(s). This information is crucial also for patients treated with CFTR modulators representing *P. aeruginosa* after prolonged negativity and to confirm chronic infections. We evaluated the ability of Fourier-transform infrared (FT-IR) spectroscopy to determine intra-patient isoclonality for 103 *P. aeruginosa* strains isolated from 36 pwCF. Two were chronically and two intermittently colonised; twelve were on modulators with a past *P. aeruginosa* colonisation; and twenty received eradication therapy, ten of whom were also treated with modulators. FT-IR data were validated by Whole Genome Sequencing (WGS) identification of Sequence Type. FT-IR identified persistence of *P. aeruginosa* in 24 patients, with a WGS-confirmed positive predictive value of 100% and diagnostic accuracy of 94%. The eradication therapy success rate was 45%, and the time to *P. aeruginosa* reappearance was similar in both patients with failed eradication treatment and those who initially cleared the infection but later acquired a new strain. Nine patients in modulators showed persistent infections. FT-IR can rapidly determine the clonality of *P. aeruginosa* isolates, allowing discrimination of recurring versus new infections both in patients with established colonisations and those subjected to eradication therapy representing *P. aeruginosa*. By overcoming the time-based criteria used to define new infections and by providing the actual success rate of eradication therapies, FT-IR can effectively contribute to the development of efficient therapeutic strategies.

## 1. Introduction

*Pseudomonas aeruginosa* is a key pathogen in cystic fibrosis (CF), associated with high morbidity and mortality in people with CF (pwCF). However, early infections generally involve wild-type, non-mucoid *P. aeruginosa* and do not appear to closely correlate with rapid lung function decline. This suggests the existence of a “window of opportunity” [1] in which early aggressive antibiotic therapy would achieve eradication of *P. aeruginosa* and postpone chronic colonisation and subsequent lung damage. Currently, early eradication therapy based on international protocols is the *de facto* standard of care for *P. aeruginosa* initial acquisition [2]. However, the success rate varies consistently based on the eradication protocol, ranging from 52 to 90% when treatment is started as soon as the bacteria are identified and genomic data are taken into consideration [3,4,5]. However, if genomic information is unavailable, the *P. aeruginosa*-free interval is generally used to evaluate whether bacterial eradication was sustained. The length of the observational period varies considerably between studies, with airways sampling completed either at the end of treatment or afterwards, resulting in non-uniform definitions [6]. Additionally, even if an eradication period is assumed to indicate the complete elimination of *P. aeruginosa,* the strain may still be present in the patient’s airways despite prolonged cultural negativity [7]. When *P. aeruginosa* reappears in sputum a few months after the treatment, it may be that bacterial eradication has not happened or reinfection occurred. This second infection can be caused either by an exogenous *P. aeruginosa* strain or by strains that spread from the sinuses to the lower respiratory tract. In clinical practice, differentiation between these two occurrences is routinely made based on time [6], possibly leading to false interpretations. Indeed, patients are considered *Pseudomonas*-free after six, and sometimes 12, months following completion of eradication therapy [4]; reappearance within these timeframes suggests that eradication therapy has failed, as demonstrated by certain studies [3]. Conversely, reappearance after more than one year suggests that a new infection with a different strain has occurred. Eradication treatment of patients with intermittent colonisation, less than 50 per cent of positive respiratory culture tests in one year for *P. aeruginosa* based on Leeds criteria [7], can also be repeated successfully [8]. This approach can help delay the onset of chronic infections virtually impossible to eradicate due to biofilm formation and mucoid strains’ appearance. Failure to clear *P. aeruginosa* from the CF lung leads to persistent colonisation, with progressive decline in lung function [4]. Therefore, determining whether the reappearing isolate has resisted therapy or whether it is a new strain is crucial for guiding therapeutic strategies to prevent chronic infection. This is also important for pwCF treated with CFTR modulators (e.g., Elexacaftor/tezacaftor/ivacaftor- ETI) representing *P. aeruginosa* after months of negativity. In these patients, *P. aeruginosa* may be isolated several months after culture negativity, possibly determined by respiratory secretions reduction caused by modulators [9]. In these cases, it is equally crucial to determine whether the reappearing isolate represents persistent colonisation or a new infection in order to set the appropriate anti-*Pseudomonas* drug therapy.

In this context, the recently developed IR Biotyper^®^ system (Bruker Daltonics GmbH, Bremen, Germany), enabling bacterial typing in 3 h with repetitions carried out over three consecutive days, offers a faster and cheaper alternative to traditional molecular techniques by providing a unique fingerprint based on the biochemical composition of cells [10,11]. Using Fourier-transform infrared (FT-IR) spectroscopy, this system distinguishes strains by quantifying the absorption of infrared light by carbohydrates, lipids, nucleic acids, proteins and lipopolysaccharides of microbial cells, generating highly specific metabolic signatures [12]. Several studies have demonstrated the applicability of FT-IR spectroscopy in place of traditional typing methods, such as Whole Genome Sequencing (WGS), due to its high discriminatory power, allowing differentiation at the species or subspecies level [11]. However, the current discriminatory power and agreement between FT-IR spectroscopy results and WGS are not always optimal. Hence, for FT-IR spectroscopy applications in areas such as epidemiological investigations and bacterial typing, it is crucial that the results obtained are consistent with WGS for phylogenetic grouping [13,14].

In an effort to move beyond the use of a *P. aeruginosa*-free interval to define bacterial eradication, we considered that bacterial eradication was achieved, and thus eradication treatment had been successful, when a reappearing strain was different from the one treated with therapy. Therefore, we used FT-IR to differentiate between new and unresolved *P. aeruginosa* infections, exploiting its potential for bacterial typing. This enabled us to monitor the efficacy of eradication treatment and the persistence of colonisation in patients treated with ETI. We compared FT-IR results with WGS to validate the discriminatory ability of FT-IR and to assess whether IR Biotyper could be a viable alternative to WGS in clinical practice for the rapid identification of clonal isolates. Enabling a complete analysis of bacterial clonality in just three days, thus fitting within the respiratory sample report turnaround time, FT-IR can provide essential information in useful time to guide clinician choice of the optimal therapeutic strategy.

## 2. Results

**Figure 1:**
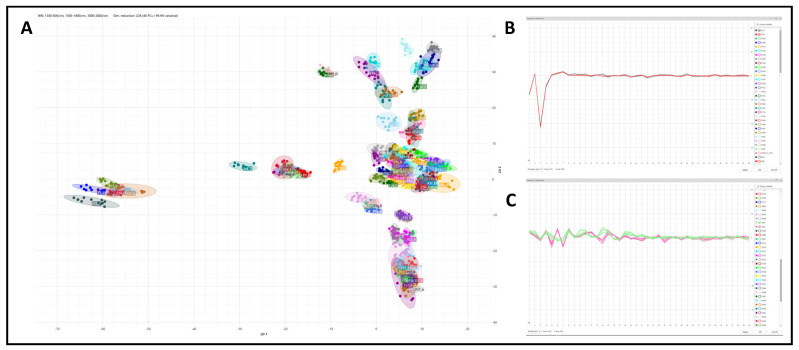
(**A**) Representation of IR Biotyper results as a 2D scatter plot (LDA with 40 principal components (PCs) accounting for 99.4% of the variation) from 103 spectra. Each dot corresponds to one spectral acquisition of each isolate; acquisitions of the same strain are the same colour and appear inside a coloured circle. (**B**) The deviation plot presents the mean spectrum of two isolates (one red, one black) along with their respective standard deviations displayed as a shaded area. (**C**) Parallel plot presents all the spectra for two isolates (one green, one pink).

Rather than relying on automated dendrogram clustering, which we found unable to identify intra-patient clonality, we determined the ‘putative clonality’ based on a 2D scatter plot (Figure 1A). To increase accuracy, we also verified the overlapping of components analysed by Linear Discriminant Analysis (LDA) in the deviation (Figure 1B) or parallel plots (Figure 1C).

Overall, the FT-IR analysis revealed putative isoclonality between at least two isolates from 24 patients. Specifically, persistence of the same *P. aeruginosa* isolate was observed in (i) two patients with chronic colonisation, (ii) two patients with intermittent colonisation, (iii) nine patients in treatment with modulators, and (iv) eleven patients undergoing eradication therapy, six of whom were also treated with modulators. Therefore, in the case of these 11 patients, FT-IR identification of persistence of the same *P. aeruginosa* following eradication therapy enabled detection of treatment failure.

Conversely, FT-IR analysis revealed evidence of successful eradication therapy in six patients, including one with a prior episode of treatment failure, and in nine additional patients also receiving modulator therapy, four of whom had experienced treatment failure either before or following the successful therapy.

Furthermore, the analysis demonstrated that four patients undergoing modulator treatment acquired new strains of *P. aeruginosa* after clearance of the original infecting clone.

**Figure 2:**
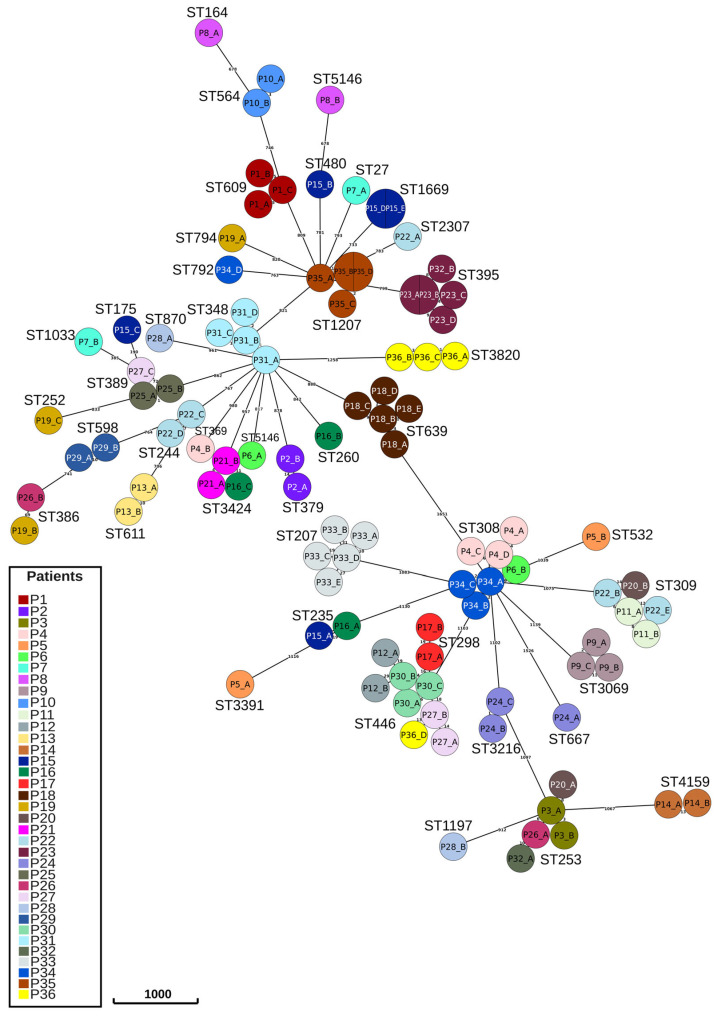
Minimum Spanning Tree (MST) of the pairwise SNP distance of strains displaying the STs they belong to. Branches display the pairwise SNP distances, while nodes report the strain ID and are coloured based on patient. Strains characterised by a 0 SNP distance are represented as a pie chart visualisation. Sequence Types (STs) the strains belong to are reported near the nodes. The MST is constructed on a core SNP of 7609 bp.

WGS was used to identify the STs of *P. aeruginosa* strains. The phylogenetic relationship between intra-patients’ isolates is presented in the Minimum Spanning Tree of Figure 2, in which genetic distance between related isolates is expressed as SNPs.

Altogether, WGS confirmed all the putative isoclonality detected by FT-IR. This confirmation comes from two factors: the identification of the same ST for patients with isoclonality and the reduced genetic distance between the isolates. The latter is indicated by the low number of SNPs, represented by the proximity of nodes in the MST (Figure 2), which shows strains from patients with isoclonality as being very close to one another.

WGS contradicted the results for four patients for whom FT-IR had identified a replacement of *P. aeruginosa* strains (Supplementary Table S1). Indeed, WGS revealed persistence of the same STs in patients P14, P18, P22 and P24. To identify possible causes of FT-IR errors, SNP differences among intra-patient isolates were determined to verify possible phylogenetic unrelatedness among the same ST isolates. Additionally, we evaluated if there were differences in morphotype (particularly the mucoid phenotype) between isolates that could explain the observed discrepancies. Differences in morphotype (Supplementary Materials) were observed in the P14 and P18 isolates. The isolates from the first patient were different from each other and not fully mucoid, while isolates from the second patient were mucoid and non-mucoid. In both P14 and P18 cases, WGS revealed a difference among intra-patient isolates between 1 and 13 SNPs (Figure 2).

**Figure 3:**
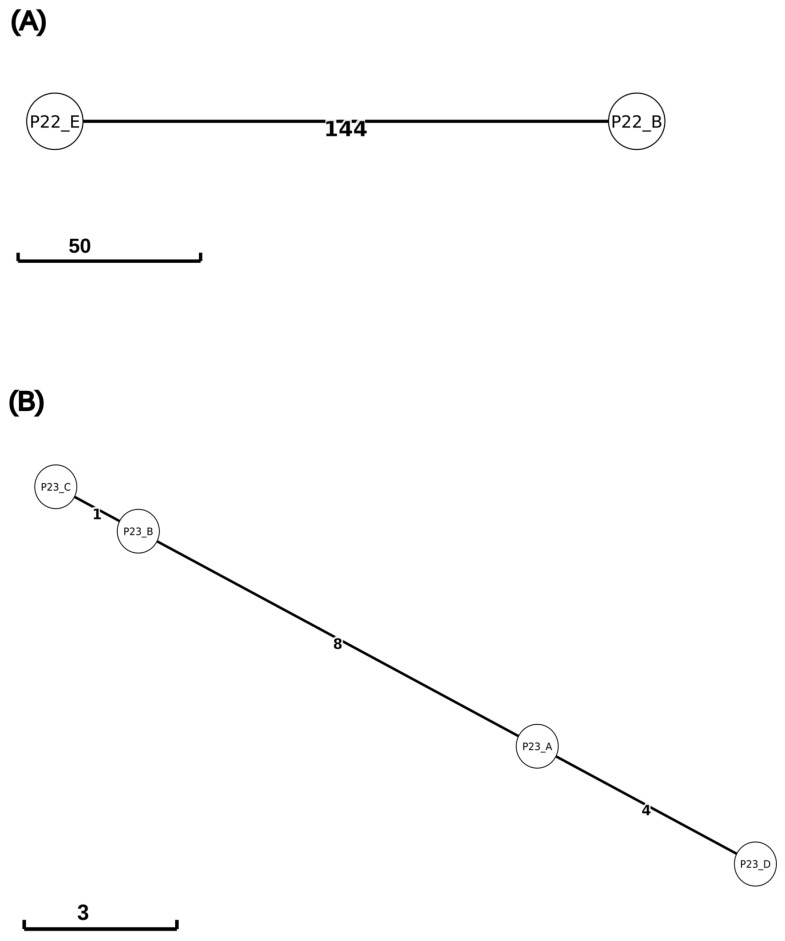
(**A**) Minimum Spanning Trees (MSTs) of the focus on the pairwise SNP distance of the P22B and P22E strains. Branches display a pairwise distance of 144 SNPs between the two isolates, suggesting that they are not the same strain. (**B**) Minimum Spanning Trees (MSTs) of the focus on the pairwise SNP distance of the P23 A–D strains. Branches display a pairwise distance of 1 SNP between P23B and P23C and 4 SNPs between P23A and P23D, suggesting that these two pairs of strains are the same strain.

In the case of P22, the two isolates P22B and P22E belong to the same ST but were identified as different from FT-IR: WGS revealed 144 SNPs of difference between the two isolates (Figure 3A), indicating that the FT-IR interpretation of unrelatedness was justified.

Discrepancies related to mucoid isolates were also observed in the FT-IR analysis of strains from patient P23, which are all mucoid and belong to the same ST (Figure 3B). These strains were found to be related in pairs: P23_A/D and P23_B/C. These pairings are supported by SNP differences between the isolates, despite them being very low. The two pairs have eight SNPs between the most proximal isolates, with P23_B and P23_C differing by one SNP, and P23_A and P23_D differing by four SNPs (Figure 3B).

No appreciable differences were found in the morphotype (not mucoid) or the number of SNPs (four) for the P24 isolates. However, both deviation and parallel plots showed high variance among the acquired spectra for each isolate as for patients P18 and P22.

Overall, when compared to WGS, FT-IR spectroscopy demonstrated a positive predictive value of 100%, confirming all identified isoclonality. However, the diagnostic accuracy was 94%, as three pairs of strains were misidentified as being unrelated. To put it another way, FT-IR appears to be a reliable method for detection of strain persistence over time, given the 100% accuracy of identifications verified by WGS. Conversely, the finding of no correlation between strains, which suggests new infections, should be treated with caution.

## 3. Discussion

*P. aeruginosa* is an opportunistic pathogen of significant clinical importance, particularly in pwCF, for whom chronic lung infections have a negative impact on the prognosis [5]. Knowledge of the clonality of *P. aeruginosa* strains isolated from the same patient over time can play a crucial role in guiding antibiotic therapy and improving clinical outcomes [6]. Indeed, the ability to detect the persistence or acquisition of a new bacterial clone makes it possible to adapt the therapeutic regimen and improve eradicating treatment efficacy.

This study demonstrates the usefulness of the IR Biotyper technology as a first-line rapid laboratory tool to determine the isoclonality of *P. aeruginosa* isolates found months apart in pwCF with repeated negative samples. By revealing *P. aeruginosa* persistence, FT-IR demonstrated that (i) two patients with intermittent colonisation are actually chronically colonised, although *P. aeruginosa* is not always isolated; (ii) nine patients under modulator are still colonised by the same ST present before the start of therapy, despite an average of 20 months of culture negativity (range 3–62 months); (iii) eradication treatment failed in five patients, indicating the necessity of a second-line therapy; and (iv) even the combination of modulator and eradication therapy was not sufficient to eradicate *P. aeruginosa* in six patients. Furthermore, FT-IR indicates that 14 patients, eight of whom were also taking modulators, probably eradicated the initial infection clones, since subsequent infections are caused by different clones. In these patients, the initial eradication treatment can be repeated to treat the new infection.

Having analysed multiple isolates from some patients, we encountered a mixture of situations, with some eradication attempts failing while others appeared to be successful within the same patient. For example, the first eradication therapy probably failed in P4, although the strain responsible for the initial infection was not detected for three years. During this time, the patient was infected by the P4_B strain, which belongs to a different ST and was apparently lost as a result of the new first-line antibiotic treatment. Conversely, P36 was infected with the same strain for over a year but successfully eradicated it after receiving second-line treatment. However, more than three years later, the patient became infected with a different strain of *P. aeruginosa*.

These results can guide therapeutic approaches’ modulation, setting second-line therapy in patients with first-line therapy failure, repeating first-line treatment in patients with a new infection, and maintaining chronic anti-*Pseudomonas* therapy in patients with intermittent infection or under a modulator. For the latter, maintenance therapy supported by isoclonality identification represents a preventive strategy that may reduce the risk of relapse and disease progression. In contrast, when FT-IR shows the emergence of a new strain in patients undergoing an eradication protocol, the result supports the repetition of a first-line eradication cycle. Therefore, applying this information to the microbiology laboratory report can benefit the patient by reducing unnecessary hospitalisation and overtreatment from second-line therapy, while increasing the chance of bacterial eradication by switching to a more aggressive treatment.

We deem critically important the observation coming from our data regarding the time intervals between isolates, especially in patients undergoing eradication treatment. Indeed, in the absence of supporting methodologies, time is currently used in clinical practice to discriminate new from persistent infections (six or twelve months). In particular, in the case of treatment failure, the mean time was 11 months (range 1–40). Conversely, disregarding extreme cases of reinfection after 5 and 13 years, patients whose *P. aeruginosa* had been successfully eradicated had a new infection after an average of 21 months (range 1–41). Therefore, even if the respective mean time seems to support the current practice, the time frames for the two events are essentially the same. This data clearly highlights the inappropriateness of distinguishing between recurring and new infections based on time, given that treatment failure can result in a positive *P. aeruginosa* culture after one year, while a new infection can occur as early as one month after the initial infection. Therefore, implementing FT-IR analysis of recurring *P. aeruginosa* infections would enable thorough evaluation of eradication protocols, prompting reflection on the therapeutic approaches adopted by each CF centre. Indeed, treatment success rate in our cohort was 45%, below what was previously reported, even in studies taking into consideration genomics in the assessment of eradication therapy efficacy [15,16]. This may be explained by the fact that, among the 20 patients who received eradication therapy in our cohort, only 13 had their first-ever *P. aeruginosa* infection during the study period. The remaining seven patients had experienced prior *P. aeruginosa* infections in the past and, because of prolonged negativity, were considered *P. aeruginosa*-free. However, our data show that in six cases bacterial eradication was only apparently achieved, meaning that we probably included cases of established colonisation in the eradication therapy success evaluation. Additionally, we should consider the possibility that ciprofloxacin and tobramycin, which are widely used in our CF centre for eradication therapy, have induced viable but non-culturable (VBNC) forms of *P. aeruginosa*. Indeed, their involvement in inducing transient and even stable forms of the VBNC stage of dormancy has indeed been demonstrated in vitro [17] and may contribute to *P. aeruginosa* infection recurrence.

Maintenance therapy using ciprofloxacin and tobramycin is also common for patients with known *P. aeruginosa* colonisation, whether intermittent or chronic. Therefore, as the possibility of these patients re-acquiring the same ST has been ruled out through SNP analysis, negative culture results may have been caused by the induction of VBNC *P. aeruginosa* or by inadequate sample production from patients who do not expectorate, even in patients with known colonisation.

One potential limitation of FT-IR in clinical practice is that it still requires WGS results’ validation, particularly when analysing pathogens such as *P. aeruginosa*, which can vary significantly in their superficial composition between morphotypes. In particular, we found it difficult to compare mucoid isolates with one another or with non-mucoid phenotypes. This hindrance, linked to the altered carbohydrate composition of such a phenotype, was only partially resolved by adding the IR lipid region to the analysis. Additionally, we observed that FT-IR analysis may lead to false interpretations when spectra from a single isolate are highly variable. This led us to mistakenly consider unrelated isolates belonging to the same ST. However, even in these cases, FT-IR was able to highlight differences that were confirmed by WGS phylogenetic reconstruction, as with P22_B and P22_E, whose unrelatedness was supported by their genomic distance. This could mean that the patient reacquired the same ST independently from the environment or that a different bacterial population reinfected the lungs, migrating from the sinuses, which are an established source of reinfection for pwCF. For this reason, we tend to disregard this interpretation as a spectrometry error, and, instead, we consider it as a reminder of the importance of considering SNPs as well as STs when obtaining confirmation through WGS.

To further complicate the analysis, CFTR modulators can significantly alter the environment of the airways, forcing *P. aeruginosa* to adapt. As a consequence of this adaptation to a new environment, the genomic and phenotypic traits of colonising *P. aeruginosa* may vary, resulting in different spectra, which further complicates comparison through FT-IR. This may have contributed to the variability of the isolates who were mistakenly interpreted as being different in the three patients.

## 4. Material and Methods

### 4.1. Bacterial Strains and Patient’s Characteristics

**Figure 4:**
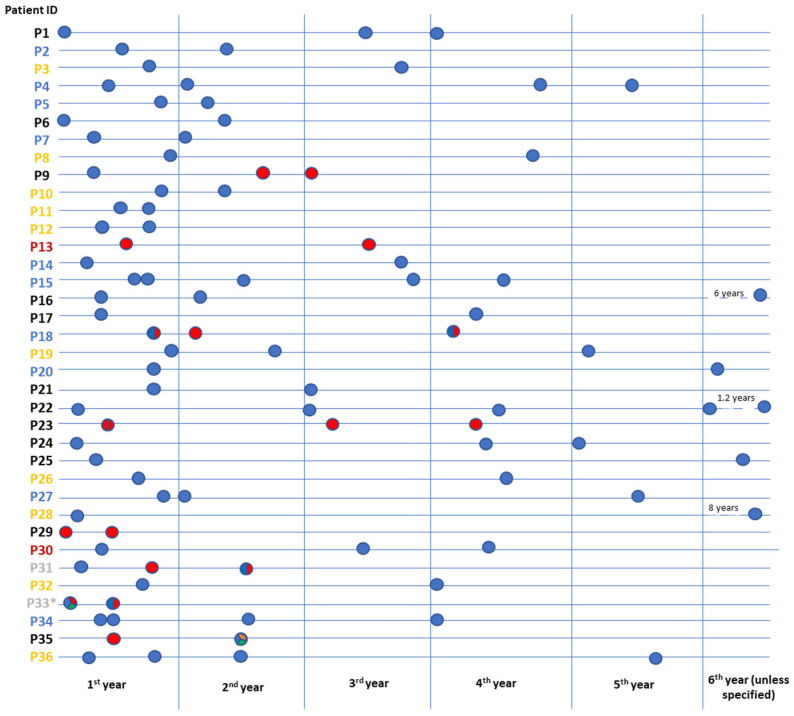
The horizontal lines represent patients, with dots indicating bacterial isolates collected over the years. The vertical lines mark the years of observation, with the dots placed in correspondence with the respective months of isolation. When two or three isolates were collected at the same time, the dot appears in two or three colours: red indicates a mucoid phenotype and blue, orange or green indicates non-mucoid isolates with different morphotypes (Supplementary Materials). Different colours are used for the patient IDs to indicate their different colonisation statuses and therapeutic interventions: black for patients treated with modulators, yellow for patients treated with eradication therapy, blue for patients treated with modulators who are also undergoing eradication therapy, red for patients with intermittent colonisation and grey for patients with chronic colonisation. *: P33 is chronically colonised and under modulator treatment.

We investigated the clonality of 103 *P. aeruginosa* isolates obtained from 36 pwCF in follow-up at the Bambino Gesù Children’s Hospital (Figure 4), who had a new positive *P. aeruginosa* sample between January 2024 and August 2025.

According to therapeutic intervention and colonisation status, defined by Leeds criteria [7], patients were classified as follows: (i) patients with intermittent *P. aeruginosa* colonisation (n = 2); (ii) patients with chronic *P. aeruginosa* colonisation, one of whom was also undergoing ETI treatment (n = 2); (iii) patients with a first *P. aeruginosa* infection and subsequent positivity following eradication therapy (n = 10); (iv) patients with a history of chronic *P. aeruginosa* colonisation and repeated negative culture results after starting modulator treatment (e.g., ivacaftor or ETI) (n = 12); and (v) patients who were treated according to eradication protocols for a new infection, either before or after starting modulator therapy (n = 10). For some patients, more than two isolates and two different time points were compared (see Figure 4).

All the 103 strains were isolated from appropriate culture media during routine microbiology monitoring of pwCF’ airways colonisation and identified by MALDI-TOF mass spectrometry. Previous isolates used to assess isoclonality were revitalised from frozen stock and kept at −80 °C.

### 4.2. FTIR Analysis

To perform IR Biotyper analysis, pure bacterial colonies were transferred to Mueller–Hinton agar (bioMérieux, Marcy l’Etoile, France). The strains were allowed to grow for 24 h at 37 °C before sample preparation. After appropriate resuspension of the colonies in 100 µL deionised water, the material was processed according to the IR Biotyper instructions. Three independent experiments (biological replicates), each involving five technical replicates, were performed to understand the variation between runs performed on three different days. The absorption of infrared (IR) light by bacterial biochemical components was measured by focusing on the IR region of carbohydrates (wavelengths between 800 and 1300 cm^−1^) and lipids (wavelengths between 400 and 1500 cm^−1^ and 2800 and 3000 cm^−1^). The first one covers most of the spectral differences between isolates of the same microbial species [18], while the second one allows for better discrimination of bacterial isolates that may vary in terms of their polysaccharide component, which is significantly modified in some phenotypes of *P. aeruginosa.* The quality of the spectra was automatically checked by evaluating the absorbance intensity, signal-to-noise ratio and water vapour interference, and all spectra that did not meet these criteria were excluded from the analysis, keeping at least 12–15 spectra for each isolate. The internal analysis criteria were established using WGS-characterised isolates as the reference standard. The FTIR analysis parameters were optimised based on the discriminatory performance observed among genetically related and unrelated isolates.

The IR Biotyper software (version 3.1.2) automatically generates a cut-off value for dendrogram-based clustering and assigns isolates with a spectral distance below the calculated threshold to the same clonal cluster. However, when isolates characterised by whole-genome sequencing (WGS) were used to set the internal analysis criteria, this approach proved to be insufficiently informative for identifying intra-patient clonality. A comparative analysis with WGS revealed that dendrogram-based clustering did not accurately reflect the spectral relationships among the isolates. Manual adjustment of the automatically generated cut-off point also failed to achieve satisfactory concordance with the WGS-defined clonal groups. Therefore, we evaluated the spectral relationships by combining the scatter plot and the deviation plot generated by the IR Biotyper software, which seemed more suitable for our objective.

The scatter plot provides a two-dimensional visualisation of the spectral relationships among isolates following dimensionality reduction by principal component analysis (PCA) and LDA. The distance between points reflects the degree of spectral similarity, evaluated with the hierarchical mean linkage clustering algorithm, which calculates the Pearson correlation coefficient of the spectra, using the instrument’s own software. During method optimisation, different LDA settings were evaluated using WGS-characterised isolates as a reference. The LDA 1–40 configuration, based on the first 40 principal components (PCs), was selected as it retained 100% of the variance in our dataset and provided the highest concordance with the WGS-defined clonal relationships.

The deviation plot shows how each analysed spectral component varies in relation to the mean spectrum of the entire dataset under evaluation. This makes it easier to identify the specific spectral regions contributing to differences between isolates. Using WGS-characterised isolates as a reference, spectral variability was evaluated within the LDA-based feature space. The most discriminatory information was typically found to be concentrated within the first ten LDA-derived PCs, which covered at least 80% of the variance. Isolates showing highly overlapping deviation patterns across these components were considered isoclonal.

Spectral similarity was calculated using the Pearson correlation coefficient implemented in the IR Biotyper software, and hierarchical clustering was performed using the average linkage algorithm.

### 4.3. WGS Analysis

WGS was used as the gold standard to identify the sequence type (ST) of the strains and validate the clonality data obtained by FT-IR spectroscopy. Genomic data were also used to reconstruct genetic distance between related isolates expressed in terms of single nucleotide polymorphisms (SNPs). This distance was used to determine whether two isolates of the same ST represented the same strain that had persisted in the patient or a new, exogenous acquisition. In particular, a cutoff of 50 SNPs was used to distinguish between the two occurrences. Bacterial DNA was extracted from pure colonies obtained from fresh samples or frozen stocks using an EZ1 automated extractor (Qiagen BioRobot EZ1, Qiagen, Hilden, Germany) with the extraction kit (EZ1&2 DNA tissue kit, Qiagen, Hilden, Germany) according to the manufacturer’s instructions. The 50 µL of extracted DNA was quantified using the Bioanalyzer instrument, and next-generation sequencing libraries were prepared using the DNA Library Prep kit (Illumina, San Diego, CA, USA) and sequenced on the Illumina MiSeq instrument (Illumina, San Diego, CA, USA) using the MiSeq Reagent Kit v3 to obtain 2 × 250 bp paired-end reads. For subsequent bioinformatics analysis, raw reads were cut for adapters and filtered for quality (Phred score > 28) using Fastp (v0.23.4, [19]), and quality was checked after cutting using FastQC (v0.11.9, [20]). Kraken2 (v2.1.3, [21]) was used to determine taxonomic classification and screen for potential contamination. Reads generated by WGS were assembled *de novo* using Shovill (v1.1.0, [22]); the quality of the assemblies was assessed using Quast (v5.1, [23]) and annotated using Prokka (v1.14.6, [24]). The Sequence Type (ST) was assessed with the MLST tool (v2.11, [25]). Single nucleotide polymorphism (SNP) calling was performed with Snippy (v4.6.0, [26]). A Minumum Spanning Tree was constructed on the core SNP alignment using Grapetree (v1.5.0).

## 5. Conclusions

Our study proves FT-IR’s ability to inform appropriate therapeutic decisions for pwCF with recurrent *P. aeruginosa* infections, showing that FT-IR had a predictive positive value of 100%, correctly identifying all cases of *P. aeruginosa* persistence. Using WGS as the gold standard and measuring the genetic distance between the isolates in terms of SNPs, we were able to determine whether the reappearance of the same ST was due to reinfection or persistence of the same strain in the lungs. This provided further evidence to support results obtained by FT-IR, which demonstrated the ability to correctly interpret strains that are genetically distant but belong to the same ST (e.g., strains from P22) as unrelated.

Despite the identified limitations, we are continuing to work towards standardising the workflow and validating the spectroscopy typing by optimising the protocol and accumulating new data. Notably, after drafting this manuscript, we analysed a further 44 strains from 14 additional patients by FTIR and sequenced them for confirmation. The PPV of FTIR was confirmed at 100%, with the diagnostic accuracy rising to 95.8%. In future, this will eliminate the need for comparison with WGS, with the exception of very limited borderline cases, which are likely to continue occurring due to the intrinsic variability of *P. aeruginosa*.

Building on previous work, our [3] study suggests revising the concept of persistence versus reinfection based solely on the time at which *P. aeruginosa* reappears after an eradication treatment course. This would prompt a revision of the definitions of *P. aeruginosa* airway infections, leading to evidence-based treatment protocols for pwCF. Furthermore, it has been previously suggested [6] that all *P. aeruginosa* strains isolated after an eradication therapy should be examined using molecular genotyping to evaluate the recurrence of the same strain. Our study suggests that a typing method that is faster and more cost-effective than molecular methods could be adopted to facilitate this assessment. This method would provide an affordable and time-efficient prediction of the clonality of intra-patient isolates that is as realistic as possible.

## Data Availability

The raw sequencing data presented in this study are deposited in the European Nucleotide Archive (ENA) repository, accession number PRJEB115747.

## Supplementary Materials

The following supporting information can be downloaded at: https://www.mdpi.com/article/10.3390/ijms27146452/s1.
